# *In situ* and in-transit analysis of cosmological simulations

**DOI:** 10.1186/s40668-016-0017-2

**Published:** 2016-08-24

**Authors:** Brian Friesen, Ann Almgren, Zarija Lukić, Gunther Weber, Dmitriy Morozov, Vincent Beckner, Marcus Day

**Affiliations:** 1 Lawrence Berkeley National Laboratory, 1 Cyclotron Road M/S 59R4010A, Berkeley, USA; 2 Lawrence Berkeley National Laboratory, 1 Cyclotron Road M/S 50A3111, Berkeley, USA; 3 Lawrence Berkeley National Laboratory, 1 Cyclotron Road M/S 50B4206, Berkeley, USA; 4 Lawrence Berkeley National Laboratory, 1 Cyclotron Road M/S 59R3103, Berkeley, USA; 5 Lawrence Berkeley National Laboratory, 1 Cyclotron Road M/S 59R4104, Berkeley, USA

**Keywords:** cosmology, post-processing, halo-finding, power spectra, *in situ*, in-transit

## Abstract

Modern cosmological simulations have reached the trillion-element scale, rendering data storage and subsequent analysis formidable tasks. To address this circumstance, we present a new MPI-parallel approach for analysis of simulation data while the simulation runs, as an alternative to the traditional workflow consisting of periodically saving large data sets to disk for subsequent ‘offline’ analysis. We demonstrate this approach in the compressible gasdynamics/*N*-body code Nyx, a hybrid $\mbox{MPI}+\mbox{OpenMP}$ code based on the BoxLib framework, used for large-scale cosmological simulations. We have enabled on-the-fly workflows in two different ways: one is a straightforward approach consisting of all MPI processes periodically halting the main simulation and analyzing each component of data that they own (‘*in situ*’). The other consists of partitioning processes into disjoint MPI groups, with one performing the simulation and periodically sending data to the other ‘sidecar’ group, which post-processes it while the simulation continues (‘in-transit’). The two groups execute their tasks asynchronously, stopping only to synchronize when a new set of simulation data needs to be analyzed. For both the *in situ* and in-transit approaches, we experiment with two different analysis suites with distinct performance behavior: one which finds dark matter halos in the simulation using merge trees to calculate the mass contained within iso-density contours, and another which calculates probability distribution functions and power spectra of various fields in the simulation. Both are common analysis tasks for cosmology, and both result in summary statistics significantly smaller than the original data set. We study the behavior of each type of analysis in each workflow in order to determine the optimal configuration for the different data analysis algorithms.

## Introduction

Data analysis and visualization are critical components of large-scale scientific computing (Ross et al. 2008; Agranovsky et al. 2014; Nouanesengsy et al. 2014; Bleuler et al. 2015; Sewell et al. 2015). Historically such workflows have consisted of running each simulation on a static compute partition and periodically writing raw simulation data to disk for ‘post-processing.’ Common tasks include visualization and size reduction of data, e.g., calculating statistics, field moments, etc. Other tasks can be domain-specific: for example, evolving large nuclear reaction networks on passively advected tracer particles in supernova simulations (Thielemann et al. 1986; Travaglio et al. 2004; Röpke et al. 2006). Often the data footprint of the output from these post-processing tasks is much smaller than that of the original simulation data (Heitmann et al. 2014; Sewell et al. 2015).

As simulations grow larger, however, this approach becomes less feasible due to disk bandwidth constraints as well as limited disk capacity. Data analysis requirements are outpacing the performance of parallel file systems, and, without modifications to either workflows or hardware (or both), the current disk-based data management infrastructure will limit scientific productivity (Ross et al. 2008; Bennett et al. 2012). One way to avoid exposure to the increasingly disparate performance of disk I/O vs. inter- and intra-node bandwidth is to limit the volume of data which is written to disk. This strategy can be realized in different ways; one approach is simply to write data relatively infrequently, e.g., every large number of time steps when evolving time-dependent problems. However, limiting the number of time steps at which grid data is saved in order to conserve disk space also discards simulation data by ‘coarsening’ in the temporal dimension (Nouanesengsy et al. 2014). For example, in order to produce a mock galaxy catalog from an *N*-body simulation, it is essential to create halo merger trees, which describe the hierarchical mass assembly of dark matter halos (Mo et al. 2010). It is, however, well recognized that in order to recover converged merger trees, identification of halos with high temporal resolution is needed (Srisawat et al. 2013).

A second strategy for addressing the I/O problem is to shift data analysis from executing ‘offline’ (on disk; Sewell et al. 2015) to running while the simulation data is still in memory. Such a workflow can be expressed in a myriad of ways, two of which we explore in this work. One approach consists of all MPI processes periodically halting the simulation and executing analysis routines on the data in memory (‘*in situ*’). The second method consists of dividing the processes into two disjoint groups; one group evolves the simulation and periodically sends its data to the other, which performs the analysis while the simulation continues asynchronously (‘in-transit;’ e.g., Bennett et al. 2012). While *in situ* approaches have long been recognized as efficient ways of avoiding I/O, less attention has been devoted to in-transit methods.

*In situ* and in-transit methods each have potential strengths and weaknesses. The former method requires no data movement beyond what is inherent to the analysis being performed. Its implementation is also relatively non-invasive to existing code bases, consisting often of adding a few strategically placed function calls at the end of a ‘main loop.’ However, if the analysis and simulation algorithms exhibit disparate scaling behavior, the performance of the entire code may suffer, since all MPI processes are required to execute both algorithms. In-transit methods, on the other hand, lead to more complex workflows and more invasive code changes, which may be undesirable (Sewell et al. 2015). They also require significant data movement, either across an interconnect or perhaps via specialized I/O accelerator (‘burst buffer’). However, they can be favorable in cases where the analysis code scales differently than that of the main simulation: since the analysis can run on a small, separate partition of MPI processes, the remaining processes can continue with the simulation asynchronously. This feature may become especially salient as execution workflows of modern codes become more heterogeneous, since different components will likely exhibit different scaling behavior.

The nature of the post-processing analysis codes themselves also plays a role in the effectiveness of in-transit implementations. In many scientific computing workflows, the ‘main’ simulation code performs a well defined task of evolving a physical system in time, e.g., solving a system of partial differential equations. As a result, its performance characteristics and science goals are relatively stationary. Analysis codes, in contrast, are implementations of a zoo of ideas for extracting scientific content from simulations. Being exploratory in nature, their goals are more ephemeral and heterogeneous than that of the simulation itself, which in general leads to more diverse performance behavior. The in-transit framework presented here provides the ability for analysis codes to be run together with the simulation, but without a strict requirement of being able to scale to a large number of cores. It is therefore useful to think of this in-transit capability as adding ‘sidecars’ to the main vehicle: in addition to resources allocated exclusively for running the simulation, we allocate a set of resources (often much smaller) for auxiliary analysis tasks.

In this work we explore both *in situ* and in-transit data analysis workflows within the context of cosmological simulations which track the evolution of structure in the universe. Specifically, we have implemented both of these workflows in the BoxLib framework, and applied them to the compressible gasdynamics/*N*-body code Nyx, used for simulating large scale cosmological phenomena (Almgren et al. 2013; Lukić et al. 2015). We test each of these workflows on two different analysis codes which operate on Nyx data sets, one which locates dark matter halos, and another which calculates probability distribution functions (PDFs) and power spectra of various scalar fields in the simulation. In Section 2 we describe the scientific backdrop and motivation for the data analysis implementations which we have tested. In Section 3 we provide the details of our implementation of the *in situ* and in-transit workflows in the BoxLib framework. Section 4 contains a description of the two analysis codes which we explored using both *in situ* and in-transit methods. We discuss the performance of the two codes in each of the two analysis modes in Section 5, and we discuss prospects and future work in Section 6.

## Cosmological simulations

Cosmological models attempt to link the observed distribution and evolution of matter in the universe with fundamental physical parameters. Some of these parameters serve as initial conditions for the universe, while others characterize the governing physics at the largest known scales (Davis et al. 1985; Almgren et al. 2013). Numerical formulations of these models occupy a curious space in the data analysis landscape: on one hand, each cosmology simulation can be ‘scientifically rich’ (Sewell et al. 2015): exploratory analysis of the simulation may lead to new insights of the governing physical model which could be lost if the raw data is reduced in memory and discarded. On the other hand, many models exhibit a highly nonlinear response to the initial perturbations imposed at high redshift, in which case isolated ‘heroic’ simulations may not capture all of the features of interest which arise from such nonlinear behavior (Davis et al. 1985; Almgren et al. 2013). Instead, one may wish to perform many such simulations and vary the initial conditions of each in order to capture the nuanced behavior of the models; such behavior can often be expressed even in a highly reduced set of data (e.g., a density power spectrum). We emphasize, then, that the data analysis methods presented here represent only a subset of techniques which will be required to manage the simulation data sets in future scales of computational cosmology.

### Formalism

The backdrop for the data post-processing methods described in this work is Nyx, a compressible gasdynamics/ *N*-body particle code for cosmological simulations of baryonic and cold dark matter (CDM) (Almgren et al. 2013; Lukić et al. 2015). Nyx characterizes the expanding universe using the Friedman equation,

1 $$ \frac{\dot{a}}{a} = H_{0} \sqrt{\frac{\varOmega _{0}}{a^{3}} + \varOmega _{\varLambda }}, $$

where $a \equiv(1+z)^{-1}$, where *z* is the redshift, $H_{0}$ is the Hubble constant, $\varOmega _{\varLambda }$ is the cosmological constant, and $\varOmega _{0}$ is the total matter content in the universe at $z=0$. The continuity equation for the baryonic gas satisfies

2 $$ \frac{\partial\rho_{b}}{\partial t} = -\frac{1}{a} \boldsymbol {\nabla} \cdot (\rho_{b} \mathbf {U}), $$

where $\rho_{b} \equiv a^{3} \rho_{\mathrm{proper}}$ is the co-moving baryon density, and **U** is the proper baryonic velocity. The baryon momentum equation is

3 $$ \frac{\partial[a \rho_{b} \mathbf {U}]}{\partial t} = -\boldsymbol {\nabla} \cdot (\rho_{b} \mathbf {U} \mathbf {U}) - \boldsymbol {\nabla} p + \rho_{b} \mathbf {g}, $$

where $p \equiv a^{3} p_{\mathrm{proper}}$ is the co-moving pressure, and **g** is the gravitational acceleration vector. Nyx uses a dual-energy formalism to evolve the baryonic energy (Bryan et al. 1995):

4 $$\begin{aligned}& \frac{\partial[a^{2} \rho_{b} e]}{\partial t} = -a \boldsymbol {\nabla} \cdot (\rho_{b} \mathbf {U} e) - a p \boldsymbol {\nabla} \cdot \mathbf {U} \\& \hphantom{\frac{\partial[a^{2} \rho_{b} e]}{\partial t} ={}}{}+ a \dot{a} \bigl[\bigl(2 - 3(\gamma-1)\bigr)\rho_{b} e\bigr] + a \varLambda _{\mathrm{HC}}, \end{aligned}$$

5 $$\begin{aligned}& \frac{\partial[a^{2} \rho_{b} E]}{\partial t} = -a \boldsymbol {\nabla} \cdot (\rho_{b} \mathbf {U} E + p \mathbf {U}) + a \rho_{b} \mathbf {U} \cdot \mathbf {g} \\& \hphantom{\frac{\partial[a^{2} \rho_{b} E]}{\partial t} ={}}{}+ a \dot {a} \bigl[\bigl(2 - 3(\gamma-1)\bigr) \rho_{b} e\bigr] + a \varLambda _{\mathrm{HC}}, \end{aligned}$$

where $E \equiv e + |\mathbf {U}|^{2}/2$ is the total energy, *e* is the internal energy, $\gamma\equiv C_{P}/C_{V}$, and $\varLambda _{\mathrm{HC}}$ represents all heating and cooling terms. This dual-energy formalism is necessary due to the numerical misbehavior inherent to hypersonic flows, where $E/e \gg1$. At the end of each time step, Nyx synchronizes *E* and *e*, the method for which is determined by their relative values. The chemical equation of state for a mixture of hydrogen and helium, as well as the heating and cooling terms in $\varLambda _{\mathrm{HC}}$, are described in Lukić et al. (2015).

The cold dark matter is treated as a pressureless, non-relativistic fluid, characterized by the Vlasov equation:

6 $$ \frac{\partial f}{\partial t} + \frac{1}{m a^{2}} \mathbf {p} \cdot \boldsymbol {\nabla} f - m \boldsymbol { \nabla} \phi\cdot\frac{\partial f}{\partial \mathbf {p}} = 0, $$

where *f* is the phase-space distribution of dark matter, *m* is its mass, **p** is its momentum, and *ϕ* is its gravitational potential. Nyx solves the Vlasov equation using a collisionless *N*-body treatment of dark matter particles. Finally, the self-gravity of the simulation is treated using

7 $$ \nabla^{2} \phi= \frac{4 \pi G}{a} (\rho_{b} + \rho_{\mathrm{dm}} - \rho_{0}), $$

where *G* is the gravitational constant, and $\rho_{0}$ the average of $\rho_{b} + \rho_{\mathrm{dm}}$ over the whole domain. Both baryonic and dark matter contribute to and are affected by the total self-gravity of the system.

Nyx is based on BoxLib, an $\mbox{MPI}+\mbox{OpenMP}$ parallelized, block-structured, adaptive mesh refinement (AMR) framework (BoxLib 2016). It evolves the two-component (hydrogen and helium) baryonic gas equations with a second-order accurate piecewise-parabolic method, using an unsplit Godunov scheme with full corner coupling (Colella 1990; Almgren et al. 2010; Almgren et al. 2013). It solves the Riemann problem iteratively using a two-shock approximation (Colella and Glaz 1985). The dark matter particles interact with the AMR hierarchy using a ‘cloud-in-cell’ method (Hockney and Eastwood 1988). The details of the numerical methods used to solve the above equations are provided in Almgren et al. (2013).

The Nyx code is fully implemented with AMR capabilities, including subcycling in time. The effectiveness of AMR has been validated in simulations of pure dark matter, as well as the ‘Santa Barbara cluster’ problem (Frenk et al. 1999; Almgren et al. 2013). However, the simulations presented in this work focus on the Lyman-*α* forest, which consists of large-scale systems in the intergalactic medium (IGM) which absorb radiation, preferentially Lyman-*α*, from distant quasars (Lukić et al. 2015). The IGM spans virtually the entire problem domain in these simulations, and as a result the signals of interest (optical depth, fluxes, etc.) are rarely localized to a specific region. As a results, it is generally impractical from a code performance perspective to use AMR at all in Lyman-*α* simulations, and as a results, all of our simulations here use a single level, with no AMR.

### Simulation data and post-processing

A typical Nyx simulation evolves a cosmology from high redshift ($z > 100$) to the present ($z = 0$) in many thousands of time steps. The size of each time step is limited by the standard CFL condition for the baryonic fluid, as well as by a quasi-CFL constraint imposed on the dark matter particles, and additionally by the evolution of the scale factor *a* in the Friedmann equation; details of these constraints are described in Almgren et al. (2013). Currently the largest Nyx simulations are run on a $4\text{,}096^{3}$ grid, and at this scale each plotfile at a single time step is ${\sim}4~\mathrm{TiB}$, with checkpoint files being even larger; a complete data set for a single simulation therefore reaches well into the petascale regime. A single simulation can therefore fill up a typical user allocation of scratch disk space ($\mathcal{O}(10)~\mathrm{TiB}$) in just a few time steps. We see then that modern simulation data sets represent a daunting challenge for both analysis and storage using current supercomputing technologies.

Nyx simulation data lends itself to a variety of popular post-processing cosmological analysis tasks. For example, in galaxies and galaxy clusters, observations have indicated that dark matter is distributed in roughly spherical ‘halos’ that surround visible baryonic matter (Davis et al. 1985). These halos provide insight into the formation of the largest and earliest gravitationally bound cosmological structures. Thus a common task performed on cosmological simulation data sets is determining the distribution, sizes, and merger histories of dark matter halos, which are identified as regions in simulations where the dark matter density is higher than some prescribed threshold. A recent review (Knebe et al. 2013) enumerates 38 different algorithms commonly used to find halos. To process data from Nyx (an Eulerian code), we use a topological technique based on iso-density contours, as discussed in Section 4.1. The approach produces results similar to the ‘friends-of-friends’ (FOF) algorithm used for particle data (Davis et al. 1985).

A second common data post-processing task in cosmological simulations is calculating statistical moments of different fields, like matter density, or Lyman-*α* flux. The first two moments - the PDF and power spectrum - are often of most interest in cosmological simulation analysis. Indeed, it is fair to say that modern cosmology is essentially the study of the statistics of density fluctuations, whether probed by photons, or by more massive tracers, such as galaxies. The power spectrum of these fluctuations is the most commonly used statistical measure for constraining cosmological parameters (Palanque-Delabrouille et al. 2013; Anderson et al. 2014; Planck Collaboration et al. 2014), and is one of the primary targets for numerical simulations. In addition to cosmology, one may be interested in predictions for astrophysical effects from these simulations, like the relationship between baryonic density $\rho_{b}$ and temperature *T* in the intergalactic medium, or details of galaxy formation.

## *In situ* vs. in-transit

Having established the scientific motivation for data post-processing in cosmological simulations, we now turn to the two methods we have implemented for performing on-the-fly data analysis in BoxLib codes.

### *In situ*

To implement a simulation analysis tool *in situ* in BoxLib codes such as Nyx, one appends to the function Amr::CoarseTimeStep() 1 a call to the desired analysis routine. All MPI processes which participate in the simulation execute the data analysis code at the conclusion of each time step, operating only on their own sets of grid data. As discussed earlier, the advantages of this execution model are that it is minimally invasive to the existing code base, and that it requires no data movement (except that inherent to the analysis itself). One potential disadvantage of *in situ* analysis is that if the analysis algorithm does not scale as well as the simulation itself, the execution time of the entire code ($\mathrm{simulation} + \mathrm{analysis}$) will suffer. Indeed, we encounter exactly this bottleneck when calculating power spectra, which we discuss in Section 4.2.

### In-transit

The in-transit implementation of data analysis codes in Nyx is more complex than the *in situ* approach, due to the necessary data movement and the asynchrony of the calculation. During initialization, BoxLib splits its global MPI communicator into two disjoint communicators, m_comm_comp for the group which executes the simulation, and m_comm_sidecar for the ‘sidecar’ group which performs the analysis. The user prescribes the sizes of each group at runtime, and the sizes are fixed for the duration of code execution. Upon reaching a time step at which analysis is requested, Nyx transfers via MPI the requisite data from the compute group to the sidecar group. Some of this data is copied to every sidecar process, e.g., the geometric information of the problem domain and how Boxes are arranged on the domain; to communicate such data, Nyx performs an intergroup MPI_Bcast(). The bulk of the data to be communicated consists of the floating-point state field stored in each Box; in BoxLib these data are called Fortran Array Boxes (FABs). Because we have two MPI groups and two communicators when executing in-transit (as well as an intergroup communicator connecting the two), we generate two ‘distribution maps’ for the simulation data, one describing the distribution of FABs across processes in the compute group, and the other in the sidecar group. This provides BoxLib with a bijective mapping of the FAB data distribution between the two groups, allowing us to perform point-to-point intergroup MPI_Send()s and MPI_Recv()s to transfer the data between corresponding processes in the two groups. We summarize the method for sending and receiving this data in Algorithm 1.

**Algorithm 1:**
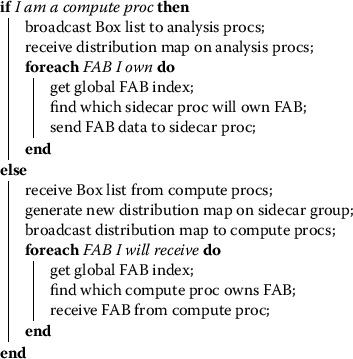
Data movement logic when sending distributed grid data from compute processes to sidecar processes via MPI.

**Figure 1:**
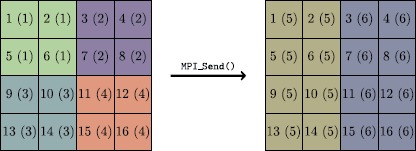
**Example schematic illustrating data movement of block-structured grids from the simulation MPI group to the sidecar group when running in-transit.** Each Box is uniquely numbered, and Boxes shaded in the same color are located on the same MPI process, whose rank is identified in parentheses. In this example, a grid composed of 16 Boxes moves from a distribution across 4 processes, with 4 Boxes per process, to a new distribution across 2 processes, with 8 Boxes per process.

The distribution of data over each MPI group need not be the same, since each group can have arbitrary size. For example, if the simulation contains 4,096 Boxes and the compute group has 2,048 processes, each process will own 2 Boxes; however, if the sidecar group has only 256 processes, each process will own 16 Boxes. After the FABs have been sent to the analysis group, that group executes the desired analysis code, while the compute group continues with the simulation. A schematic of this Box movement across MPI groups is depicted in Figure 1.

The receipt of FABs onto a single MPI process is an inherently serial process. This property can affect code performance adversely if a large number of compute processes send their data to a small number of sidecar processes, because a compute process cannot continue with the simulation until all of its FABs have been sent, and each sidecar process can receive only one FAB at a time. In the example shown in Figure 1, process 5 receives FABs from processes 1 and 3; if, by coincidence, process 5 receives all four of process 3’s FABs in order, process 3 can continue with its next task before process 1; however, process 5 cannot continue until it has received all FABs from both processes 1 and 3. In this example the ratio of sending to receiving processes, $R \equiv N_{s} / N_{r} = 2$, is relatively small; the serialization of this data transfer will have only a minor effect on aggregate code performance. However, if $R \sim\mathcal{O}(100)$ or $\mathcal{O}(1\text{,}000)$, the effect will be more pronounced.

### Task scheduling

In both the *in situ* and in-transit workflows in BoxLib, we have added a simple queue-based, first-in-first-out scheduler which governs the order of data analysis tasks being performed. As we generally have a small number of tasks to perform during analysis, this approach is quite satisfactory. If the number of analysis tasks grows larger (a trend which we expect), then the workloads of each of these tasks will become more complex, and the variability in scaling behavior of each may be large as well. In this case a more sophisticated scheduling system - in particular one which accounts for a heuristic describing the scalability of each task and allocates sidecar partitions accordingly - may become more useful.

## Cosmological simulation analysis tools

Many types of cosmological simulations can be broadly characterized by a small set of quantities which are derived (and highly reduced) from the original simulation data set. Such quantities include the distribution and sizes of dark matter halos, PDFs and power spectra of baryon density, dark matter density, temperature, Lyman-*α* optical depths, etc. (Lukić et al. 2015). We obtain these quantities from Nyx simulation data using two companion codes: Reeber, which uses topological methods to compute dark matter halo sizes and locations; and Gimlet, which computes statistical data of various fields. Because the algorithms in these codes are very different, the codes themselves exhibit different performance and scaling behavior. Therefore, they together span a useful parameter space for evaluating the utility of *in situ* and in-transit methods. In addition to operating on data in memory, both Reeber and Gimlet are capable of running ‘offline,’ in the traditional post-processing workflow described in Section 1. We describe each of these codes below.

### Reeber

Reeber is a topological analysis code, which constructs merge trees of scalar fields. A merge tree describes the relationship among the components of super-level sets, i.e., regions of the data set with values above a given threshold. The leaves of a merge tree represent maxima; its internal nodes correspond to saddles where different components merge; its root corresponds to the global minimum (Morozov and Weber 2013). In the case of Reeber operating on Nyx data sets, the points *x* and *y* correspond to distinct grid points $\mathbf {r}_{1}$ and $\mathbf {r}_{2}$, and the function *f* corresponds to the density $\rho(\mathbf {r})$.

**Figure 2:**
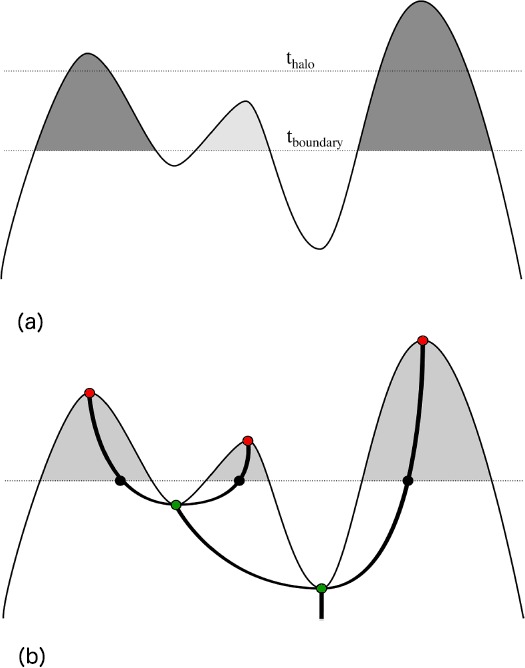
**Illustration of merge trees and their relationships to isosurfaces.** The top subfigure **(a)** illustrates the halo definition based on iso-density contours. Halos are regions above a density threshold $t_{\text{boundary}}$ (light gray region) whose maximum density exceeds $t_{\text{halo}}$ (dark gray regions). The bottom subfigure **(b)** shows the merge tree which corresponds to the level set parameters $t_{{\text{boundary}}}$ and $t_{{\text{halo}}}$ given in Figure 2(a). The black dots correspond to points on the same super-level set, with each representing a different connected component on that super-level set. The green dots indicate saddle points of the scalar function, while the red dots indicate local maxima.

An illustration of Reeber’s halo-finding algorithm is shown in Figure 2(a). To identify iso-density-based halos efficiently, we traverse the merge tree upwards from the root (i.e., from the global minimum density), finding all edges that cross the value $t_{\mathrm{boundary}}$. This operation corresponds to drawing the merge tree such that the height of each node corresponds to its function value and identifying all sub-trees above a line at the height of $t_{\mathrm{boundary}}$. We then traverse each sub-tree to identify its highest maximum. If this maximum exceeds $t_{\mathrm{halo}}$, the sub-tree represents a halo, and we compute its position as the centroid of all grid points belonging to the sub-tree as well as its mass as the cell-size-weighted sum of all grid points belonging to the sub-tree. The merge tree corresponding to the halo-finding parameters in Figure 2(a) is illustrated in Figure 2(b).

**Figure 3:**
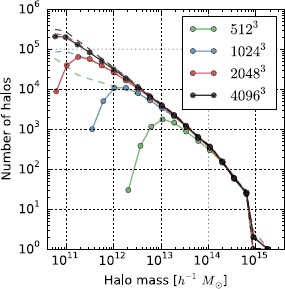
**Convergence of the halo mass function in Nyx simulations with the Reeber halo finding code.** Solid lines demonstrate how Reeber’s distribution of halo masses change when increasing the spatial resolution in Nyx runs. As expected, we observe the differences only at the low-mass end, since coarse grids cannot capture well small halos, while the agreement on the high-mass end is good. The dashed lines show results of a FOF halo finder when the linking length parameter is chosen to match approximately the iso-density contour used in Reeber. FOF results are used as a validation here, showing that Reeber results converge to the ‘correct’ answer.

As a topological descriptor, a merge tree is primarily a means to an end: its utility comes from providing an efficient way to query a field for interesting topological information, e.g., are two regions of a field connected above a given threshold, or what is the number of connected components at density *ρ* whose density maximum is higher than $\rho'$? In Nyx simulations, one requires the answer to exactly these questions when identifying the locations, distribution, and merger histories of dark matter halos, as discussed in Section 2. Given the dark matter density field and its boundary conditions, Reeber first constructs its merge tree, and then uses it to identify halos based on user-defined density thresholds (Morozov et al., in preparation). The merge tree itself does not depend on any parameters, only on the input function. Accordingly, one can repeatedly query the same tree with different halo thresholds without reconstructing it each time. A recent result applying Reeber to various Nyx simulation data sets is shown in Figure 3.

Given that so many models and methods for generating halo mass functions already exist (Knebe et al. 2013), it is prudent to validate the iso-density approach used by Reeber by comparing with others. We have found close agreement with the FOF halo mass function (see Figure 3), although the two approaches for finding halos are quite different. Even more useful for comparison would be analytic or semi-analytic halo mass function results, such as the Press-Schechter formalism (Press and Schechter 1974); however, as shown in Lukić et al. (2009), the FOF model and the spherical collapse model used in the Press-Schechter function are incompatible.

Implementing a scalable representation of merge trees on distributed memory systems is a challenging but critical endeavor. Because the merge tree is a global representation of the entire field, traditional approaches for distributing the tree across independent processes inevitably require communication-intensive reduction to construct the final tree, which in turn lead to poor scalability. Furthermore, modern simulations operate on data sets that are too large for all topological information to fit on a single compute node. Reeber’s ‘local-global’ representation addresses this problem by distributing the merge tree, so that each node stores detailed information about its local data, together with information about how the local data fits into the global merge tree. The overhead from the extra information is minimal, yet it allows individual processors to globally identify components of super-level sets without any communication (Morozov and Weber 2013). As a result, the merge trees can be queried in a distributed way, where each processor is responsible for answering the query with respect to its local data, and a simple reduction is sufficient to add up contribution from different processes. A detailed description of merge trees, contour trees, and their ‘local-global’ representation, which allows Reeber to scale efficiently on distributed memory systems, is given in Morozov and Weber (2013), Morozov and Weber (2014) and its application to halo finding in Morozov et al. (in preparation).

### Gimlet

Gimlet calculates a variety of quantities relevant for the intergalactic medium studies, which are derived from different fields in Nyx, including:

- optical depth and flux of Lyman-*α* radiation along each axis
- mean Lyman-*α* flux along each axis
- 2-D probability distribution function (PDF) of temperature vs. baryon density
- PDF and power spectrum of Lyman-*α* flux along each axis
- PDF and power spectrum of each of
  - baryon density
  - dark matter density
  - total matter density
  - neutral hydrogen density

We calculate the optical depth *τ* of Lyman-*α* radiation in the optically thin limit (Lukić et al. 2015):

8 $$ \tau_{\nu}\equiv \int dr n_{X} \sigma_{\nu}\simeq\frac{\pi e^{2}}{m_{e} c} f_{12} \int dr \frac{n_{X}}{\varDelta \nu_{D}} \frac{\exp [- ( \frac {\nu- \nu_{0}}{\varDelta \nu_{0}} )^{2} ]}{\sqrt{\pi}}, $$

where $n_{X}$ is the number density of neutral hydrogen, $\sigma_{\nu}$ is the opacity to Lyman-*α* radiation, *e* is the elementary charge, $m_{e}$ is the electron mass, *c* is the speed of light, $f_{12}$ is the oscillator strength of the Lyman-*α* transition, $\varDelta \nu_{D} \equiv(b/c) \nu_{0}$ is the Doppler width of the Lyman-*α* transition with the Doppler parameter $b = b_{{\mathrm{thermal}}} \equiv \sqrt{2 k_{B} T/m_{H}}$, *T* is the gas temperature, and $m_{H}$ is the mass of the hydrogen atom. In the optically thin limit, and absent scattering effects, *τ* becomes a purely local quantity (Mihalas 1978). Similarly, the flux due to Lyman-*α* radiation is also a local quantity, being a simple rescaling of the neutral hydrogen number density $n_{X}$ (Lukić et al. 2015):

9 $$ F_{\nu}= \exp(-\tau_{\nu}). $$

The most interesting quantities calculated by Gimlet are power spectra of the Lyman-*α* flux and matter density. Gimlet uses FFTW3 (Frigo and Johnson 2005) to calculate the discrete Fourier transformation (DFT) of the grid data required for power spectra. We note here also that FFTW3’s domain decomposition strategy for 3-D DFTs has implications which are especially relevant for a study of *in situ* and in-transit analysis performance. We discuss these in Section 5.

Given a 3-D scalar field, Gimlet calculates its DFT in two different ways:

1. It divides the grid into one-cell-thick columns which span the length of the entire problem domain. All columns are aligned along one of the 3 axes. It then compute the 1-D DFT and along each column individually, accumulating the results into a power spectrum for the entire grid. This approach captures line-of-sight effects of the cosmology simulation, as discussed in Lukić et al. (2015). Each DFT can be executed without MPI or domain decomposition, since the memory footprint of each column is small, even for large problem domains.
2. It computes the 3-D DFT and power spectrum of the entire grid. This requires exploiting FFTW3’s domain decomposition features enabled with MPI.

**Figure 4:**
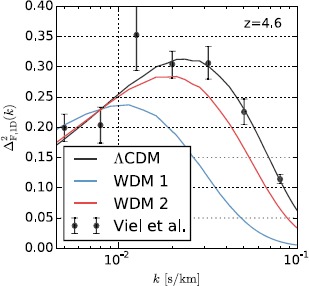
**Power spectrum of Lyman-** ***α*** **flux from 3 Nyx simulations using the Gimlet analysis code, compared to observational data presented in Viel et al. (** 2013 **).** The black line is the result of a *Λ*CDM cosmological model with the reionization history described in Haardt and Madau (2012). The blue and red lines are two WDM models, differing in their choice of dark matter particle mass: $m_{\text{DM}} = 0.85~{\text{keV}}$ (blue) and $m_{\text{DM}} = 2.1~{\text{keV}}$ (red).

As an example of Gimlet application, we show in Figure 4 a comparison between the observed Lyman-*α* flux power spectrum, and predictions from 3 Nyx simulations. We plot a dimensionless power spectrum calculated along the line of sight versus the wavelength mode *k*. We show here one redshift only using data from Viel et al. (2013), and we demonstrate how power spectra differ when changing the thermal velocity dispersion of the dark matter. The black line is the cold dark matter model which has no thermal velocity component in the initial state. The blue and red lines correspond to two different warm dark matter (WDM) models, $m_{\mathrm{DM}} = 0.85~{\mathrm{keV}}$ and $m_{\mathrm{DM}} = 2.1~{\mathrm{keV}}$, respectively. The main task of Nyx simulations with the Gimlet analysis pipeline is to determine which cosmological model and reionization history fits the best existing observational data, and Figure 4 is a simple example when we vary only one parameter out of ∼10.

The two basic types of calculations Gimlet performs - PDFs and power spectra - exhibit quite different performance behavior. PDFs scale well, since each MPI process bins only local data. A single MPI_Reduce() accumulates the data bins onto a single process, which then writes it to disk. Power spectra, on the other hand, require calculating communication-intensive DFTs. The need to reorganize data to fit domain decomposition required by the FFTW3 library - which differs from the native Nyx decomposition - incurs additional expense. Gimlet’s overall scalability, therefore, is a convolution of these two extremes.

## Performance

In this section we examine detailed performance figures for the *in situ* and in-transit workflows described above. All of these tests were performed on Edison, a Cray XC30 supercomputing system, at the National Energy Research Scientific Computing Center (NERSC). Edison’s compute partition consists of 5,576 nodes, each configured with two 12-core Intel Xeon ‘Ivy Bridge’ processors at 2.4 GHz, and 64 GB of DDR3 memory at 1,866 MHz. Compute nodes communicate using a Cray Aries interconnect which has ‘dragonfly’ topology. The high-performance scratch file system is powered by Lustre, and has a total capacity of 2.1 PB (1.87 PiB) and a peak bandwidth of 48 GB/s (44.7 GiB/s), distributed across 96 object storage targets (OSTs).

First we present results of some synthetic performance benchmarks, in order to establish baselines by which to compare the performance of real analyses performed by the Reeber and Gimlet. Then we present the results from the analysis codes themselves. We note that, although Nyx and other BoxLib-based codes use OpenMP to express *intra*-node parallelism, we disabled OpenMP for all performance tests presented below (in Nyx, Gimlet, and Reeber), since the focus of this work is primarily on *inter*-node relationships. Furthermore, because both the Box size and the total problem domain size of Nyx simulations is typically a power of 2, we use only up to 8 MPI processes per socket on an Edison compute node, always with only 1 MPI process per core. The extra 4 cores on each socket generally do not expedite the computation significantly, both due to load imbalance (12 and 24 rarely divide evenly into the problem domain size), as well as to the fact that, even with only 8 out of 12 cores active on a given socket, we already saturate most of the available memory bandwidth due to the low arithmetic intensity of the finite-volume algorithms in Nyx (Williams et al. 2009). Again, we applied this power-of-2 rule to the Gimlet and Reeber codes as well.

### Lustre file write performance

*In situ* and in-transit methods attempt to circumvent the limitations of not only disk capacity, but also disk bandwidth. It is therefore of interest to this study to measure the time a code would normally spend saving the required data to disk in the traditional post-processing workflow. To do so, we measured the time to write grids of various sizes, each containing 10 state variables,^2^ to the Lustre file system on Edison. BoxLib writes simulation data in parallel using std::ostream::write to individual files; it does not write to shared files. The user specifies the number of files over which to distribute the simulation data, and BoxLib in turn divides those files among its MPI processes. Each process writes only its own data, and only one process writes to a given file at a time, although a single file may ultimately contain data from multiple processes. The maximum number of files allowable is the number of processes, such that each process writes its own data to a separate file.

We varied the size of the simulation grid from 128^3^ to $2\text{,}048^{3}$, divided among Boxes of size 32^3^. This is a small Box size for Lyman-*α* simulations which typically do not use mesh refinement; however, it is entirely typical for many other BoxLib-based applications which perform AMR. The maximum number of processes for each test was 8,192, although for tests which had fewer than 8,192 total Boxes (namely, those with 128^3^ and 256^3^ simulation domains), we set the number of processes such that each process owned at least 1 Box. We also varied the number of total files used, from 32 up to 8,192, except in cases where there were fewer than 8,192 total Boxes.

**Figure 5:**
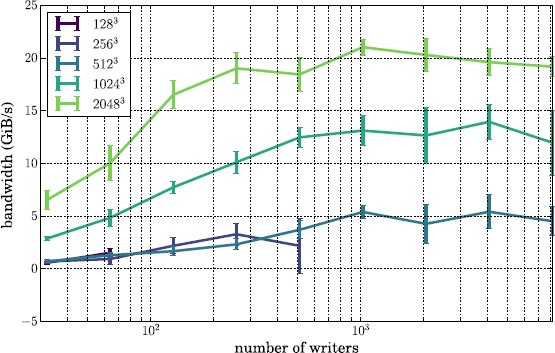
**Aggregate bandwidth (GiB/s) for writing 10-component simulation grids of varying sizes to Lustre.** Each data point shows the statistical mean over 5 writes, with the standard deviation shown in error bars. The lack of data points for the 128^3^ and 256^3^ grids at large numbers of writers are configurations with more MPI processes than total Boxes, such that some portion of processes would write no data.

We illustrate the file write performance on Lustre in Figure 5. We find that, for the largest grid tested ($2\text{,}048^{3}$), the highest achievable write bandwidth on the Edison Lustre file system is ${\sim} 20~\mathrm{GiB}\mbox{/}\mathrm{s}$, about $(\sim)45\%$ of the peak bandwidth available on this file system. For the 512^3^ grid, which serves as our test case when we explore the performance of MPI traffic when running Nyx analysis codes in-transit in Section 5, the highest write bandwidth is ${\sim}5~\mathrm{GiB}\mbox{/}\mathrm{s}$, about 11% of peak bandwidth.

### In-transit MPI performance

**Figure 6:**
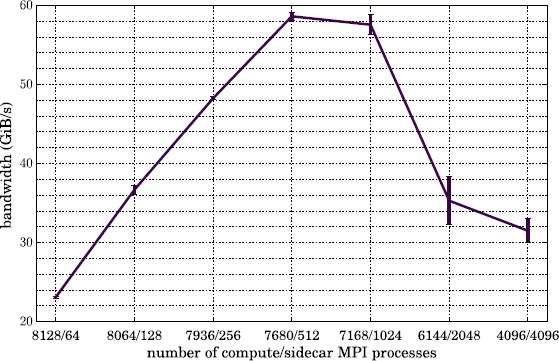
**Total bandwidth during transfer of a 10-component** $\pmb{1\text{,}024^{3}}$ **grid (32,768 Boxes) among 8,192 total MPI processes, with varying sizes of compute and analysis groups.** The standard deviation over 5 iterations is indicated with error bars.

**Figure 7:**
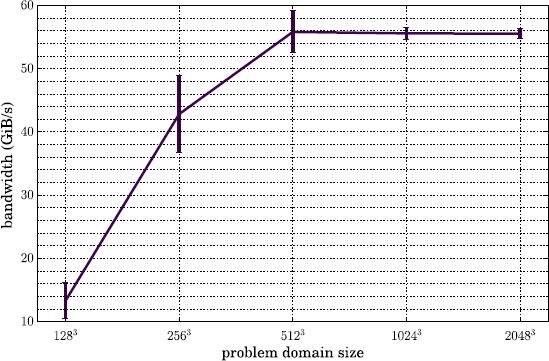
**Total bandwidth during transfer of 10-component grids of varying sizes from 7,168 compute MPI processes to 1,024 analysis MPI processes.** The standard deviation over 5 iterations is indicated with error bars.

Before exploring the analysis workflow performance study which is presented in Section 5.4, here we first perform two simple studies which measure the time required to move grid data from one MPI group to another. In one test we used a 10-component grid of size $1\text{,}024^{3}$, which has a total memory footprint of ${\sim}80~\mathrm{GiB}$. The grid was divided into Boxes of size 32^3^, yielding 32,768 total Boxes. We then fixed the total number of MPI processes at 8,192, and varied the number of analysis processes from 64 to 4,096 (with the size of the compute group varying from 8,128 to 4,096 processes). The results for this test are shown in Figure 6. In the second test, we fixed the number of total processes at 8,192 and also fixed the number of analysis processes at 1,024, leaving 7,168 processes in the compute group. We then varied the size of the grid to be transferred from 128^3^ (64 Boxes with total size 156 MiB) to $2\text{,}048^{3}$ (262,144 Boxes with total size 640 GiB). The results are presented in Figure 7.

We see from this figure that the fastest bandwidth we achieve across the interconnect is ${\sim}58~\mathrm{GiB}\mbox{/}\mathrm{s}$. The peak bandwidth for the entire Aries interconnect on Edison is 23.7 TB/s (${\sim}21.6~\mathrm{TiB}\mbox{/}\mathrm{s}$) distributed across 5,576 compute nodes, indicating a peak bandwidth per node of ${\sim}4.0~\mathrm{GiB}\mbox{/}\mathrm{s}$. From our test, the highest bandwidth per node was ${\sim}116~\mathrm{MiB}\mbox{/}\mathrm{s}$, only about 3% of peak. Since several different configurations in this test appear to plateau at the same bandwidth, this limit may be due to high latency costs associated with sending and receiving so many small Boxes. Were the Boxes much larger, as they often are in Lyman-*α* simulations, the MPI traffic would consist of fewer and larger messages, which may achieve higher per-node bandwidth. However, even performing so far below the peak, the bandwidth of moving Boxes across the interconnect for the 512^3^ grid is still a factor of 10 faster than the fastest bandwidth achieved saving it to disk on the Edison Lustre file system (${\sim}45\%$ of peak; cf. Figure 5).

From this simple study we can estimate that, in terms of pure transfer speed (neglecting analysis or simulation scaling behavior), the optimal ratio of compute to analysis on Edison is $R \sim9$. For analysis algorithms which strong scale efficiently, this should be a useful metric. The poor bandwidth for the small grids in Figure 7 arises because there are more MPI processes than Boxes, so many processes do not participate in the transfer process; the simulation therefore has access to a smaller portion of the aggregate available bandwidth on the interconnect. In all cases, the total transfer time is on the order of seconds. If analysis is performed infrequently, this comprises a small component of the total run time of most Nyx simulations, which often take ${\gtrsim}\mathcal{O}(10^{5})~\mathrm{s}$. However as the frequency of analysis increases, the total time spent moving data can become considerable, and an *in situ* approach may become more attractive.

The complexity of in-transit analysis presents a number of factors one should consider in order to optimize code performance. For example, the cost of moving large volumes of data across MPI groups via an interconnect is significant, but can nevertheless be dwarfed by the cost of the analysis computation itself. Additionally, some analysis algorithms scale poorly - significantly worse than the simulation - in which case incurring the penalty for moving data to a small set of sidecar processes so that the simulation can continue may lead to the best overall performance of the code. On the other hand, the data movement penalty makes in-transit data processing impractical for applications which are already inexpensive to calculate, or which scale very well, or both. These types of analysis may be more amenable to *in situ* approaches.

In-transit analysis introduces an additional layer of load balancing complexity, in that an optimally performant simulation maximizes the asynchrony of computation and analysis. To illustrate this point, suppose a simulation for evolving a system forward in time reserves *C* MPI processes for computation and *A* for analysis. Denote by $\tau_{c}$ the time required for the compute processes to complete one time step of the simulation (without analysis), and $\tau _{a}$ the time for the analysis processes to complete analysis for one set of data. If the user requests that the analysis execute every *n* time steps, then an optimal configuration of compute and analysis groups would have $n \tau_{c} \simeq\tau_{a}$. If the former is larger, then the analysis group finishes too early and has no work to do while it waits for the next analysis signal; the converse is true if the latter is larger. If one finds that $n \tau_{c} > \tau_{a}$, then one option is to decrease *A*, the number of analysis processes. This will increase the $\tau_{a}$ but will simultaneously decrease $\tau _{c}$ since we assume that $C + A = \mathrm{const}$. A second option to equilibrate the two time scales is to decrease *n*, although this option may not be useful in all cases, since *n* is likely driven by scientific constraints and not code performance. If one relaxes the restriction that $C + A = \mathrm{const.}$, then one can adjust the size of the analysis group arbitrarily while keeping the compute group fixed in order to balance the two time scales $\tau_{c}$ and $\tau_{a}$.

The risk of load imbalance described above traces its roots to the static nature of the roles of compute and analysis processes which we have assumed for this example, and which has traditionally been characteristic of large-scale simulations. Much of it could be ameliorated using dynamic simulation ‘steering,’ in which the simulation periodically analyzes its own load balance and adjusts the roles of its various processes accordingly. Returning to the above example, one may request an additional task from the analysis group: every *m* time steps, it measures the time spent in MPI calls between the compute and analysis groups (such calls remain unmatched while one group is still working). If $n \tau_{c} > \tau_{a}$, then before the next time step the simulation re-sizes the MPI groups to increase *C* and decrease *M*, by an amount commensurate with the length of the time spent waiting for both groups to synchronize. An even more exotic solution would be to subsume *all* resources into the compute group until the *n*th time step, at which point the simulation spawns the analysis group on the spot, performs the analysis, and re-assimilates those processes after analysis is complete. Another possibility would be to decompose the tasks of simulation evolution and post-processing *within* a node, using OpenMP, for example. This may alleviate the data movement penalty, requiring data to move only across NUMA domains within a compute node (or perhaps not at all), the speed of which would be significantly faster than moving across the interconnect. The disadvantage of this approach would be that *all* codes involved in the simulation/post-processing workflow would need to contain a hybrid $\mbox{MPI}+\mbox{OpenMP}$ implementation; for codes which are old or large, or which have a large ecosystem of already existing ‘companion’ codes for post-processing, this can be a laborious process. If OpenMP is not an option for controlling process placement on compute nodes, some MPI libraries provide mechanisms for placing processes on nodes by hand, although we are unaware of any truly portable method of doing so. The heuristics used to adjust the simulation configuration will likely be problem-dependent and will need to draw from a statistical sample of simulation performance data; we are actively pursuing this line of research.

### Problem setup

Having established some synthetic benchmarks for disk and interconnect bandwidths, we now turn to the application of these workflows on science problems in Nyx. Here we present an exploratory performance analysis of *in situ* and in-transit implementations of both Reeber and Gimlet. We evolved a Lyman-*α* forest problem (Lukić et al. 2015) uniform grid (no mesh refinement) for 30 time steps, resuming a previous simulation which stopped at redshift $z \sim3$. We chose this cosmological epoch because this is often when the most ‘post-processing’ is performed, due the wealth of observational data available for comparison (Heitmann et al. 2015; Lukić et al. 2015). The domain boundaries were periodic in all three dimensions. We ran the simulation in three different configurations: with no analysis, with Reeber, and with Gimlet.^3^ In simulations which perform analysis, the analysis code executed every 5 time steps, starting at step 1. We choose this configuration such that the last time step during which analysis is performed is number 26; this gives the sidecar group a ‘complete’ window of 5 simulation time steps (26-30) in which to perform the last analysis. Finally, we repeated this calculation on two different grid sizes, 512^3^ and $1\text{,}024^{3}$, in order to explore the performance of these algorithms at different scales.

When running *in situ*, we ran the simulation and analysis on a fixed number of MPI processes (2,048 processes for the 512^3^ problem and 4,096 for the $1\text{,}024^{3}$ problem). For the in-transit mode, we chose the number of MPI processes in two different ways. First, we fixed the number of *total* processes at the same number used for the *in situ* run, and varied the number of processes allocated to either the simulation or sidecars. This approach shows the optimal balance of each if the goal is to fit the simulation into a desired queue on a computational system which has a restriction on the maximum number of total processes. In the text which follows, we label this configuration ‘CT.’ Our second approach was to fix the number of processes devoted to the *simulation* at the same number used for *in situ*, and to vary the number of *additional* processes devoted to analysis. The total number of processes was then larger than the number used for the *in situ* run. This approach has in mind the case that the user wishes for the grid data to be distributed among the simulation cores in a particular way to preserve load balance, and that one is less concerned with the total number of processes being used. We denote this configuration as ‘CS.’ The details of each configuration are listed in Table 1. An example illustrates the influence of load imbalance across MPI processes: if we have 4,096 Boxes spanning the domain but only 2,047 processes evolving the simulation instead of 2,048, two of those 2,047 process must each operate on 3 Boxes, while the other 2,045 processes operate on only 2. However, *all* processes must wait for the two processes which are computing 3 Boxes. Thus, decreasing the computational resources by 0.05% increases the total run time by 50%.

**Table 1:** **Summary of problem configurations used for performance analysis of post-processing implementation in BoxLib**

Grid size	512^3^	1,024^3^
Problem size	10 Mpc	20 Mpc
Resolution	${\sim}20~\mbox{kpc}$	${\sim}20~\mbox{kpc}$
Box size	64^3^	128^3^
# Boxes	4,096	4,096
# MPI procs *in situ*	2,048	4,096
# MPI procs (CT)	2,048	4,096
# MPI simulation procs (CS)	2,048	4,096

‘CT’ denotes the total number of MPI processes used in-transit, whereas ‘CS’ is the number of processes devoted purely to evolving the simulation, with an additional set of processes dedicated to performing post-processing (see Section 5.3 for a complete description of these configurations).

In all simulations, we ran Nyx, Reeber, and Gimlet using pure MPI, with no OpenMP. We used version 3.3.4.6 of FFTW3, and compiled all codes with the GNU compiler suite, version 5.2.0, with ‘-O3’ optimization. In all DFT calculations, we used FFTW3’s default ‘plan’ flag, FFTW_MEASURE, to determine the optimal FFTW3 execution strategy.

### Results

**Figure 8:**
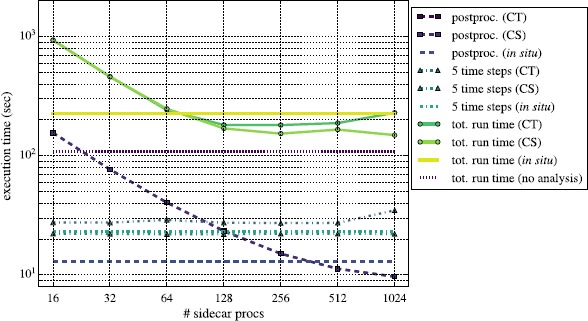
**Performance of Reeber running** ***in situ*** **and in-transit with different distributions of MPI processes on a** $\pmb{512^{3}}$ **problem.** The times indicated are wall clock seconds. We used two different in-transit configurations: once with a constant total number of 2,048 MPI processes (‘CT’), and once with a constant number of processes (2,048) devoted to simulation (‘CS’). Time to post-processes for the CS and CT in-transit configurations are nearly identical. The short-dashed purple line indicates the Nyx run time with 2,048 MPI processes without performing any analysis.

We summarize our performance results in Figures 8 through 11. There we compare various components of the simulation running both Reeber and Gimlet *in situ* and in-transit. Specifically, we plot the total end-to-end run times in solid lines, the time for each post-processing step (every 5th time step) as dashed lines, and the time to evolve the simulation 5 time steps with dot-dashed lines. For visual clarity, the in-transit runs have markers at each data point, while the *in situ* lines are unmarked, as they represent a single data point. Each data point for the post-processing execution time is an average over 6 iterations; the standard deviations illustrated with error bars would be smaller than the line thickness and are thus not indicated in either figure. Each total run time is a single data point since each complete configuration was run only once. The lines labeled ‘CT’ used a constant number of *total* processes, such that, e.g., when using 2,048 total processes with 16 sidecar processes, the remaining 2,032 are running the simulation. Those labeled ‘CS’ have a constant number of processes working on the simulation alone, such that when using 16 sidecar processes and additional 2,048 for evolving the simulation, a total of 2,064 processes are running. The *in situ* lines are horizontal and independent of the number of sidecar processes; they use a fixed number of 2,048 total processes, all of which perform both simulation and analysis. The CT and *in situ* configurations, therefore, always use the same number of total processes, while the CS configurations use more. We also plot with the short-dashed purple line the total time to run the Nyx simulation without any analysis, running on 2,048 processes.

The times to post-process (dashed lines in each of the four figures) illustrate the strong scaling behavior of both analysis codes. The interplay among the different scalabilities presented here - those of the analysis suite, simulation code itself, and the combination of the two - are complex and require a nuanced interpretation. In Figure 8 we see that Reeber strong scales efficiently, although its performance begins to plateau at large numbers of MPI processes, due to the relatively small problem size (a 512^3^ problem domain). We also note that Reeber running *in situ* on 2,048 processes is actually slower than on 512 or 1,024 processes in-transit, which is due to having too many processes operating on too small a problem.

Figure 8 also illustrates the relationship between the scalability of the analysis code alone and that of the entire code suite (simulation+analysis). In particular, we see that for ≤64 sidecar processes performing analysis, a single Reeber analysis call takes longer than the 5 intervening simulation time steps. As a result, the strong scaling behavior of the complete simulation+analysis suite mirrors that of Reeber almost exactly. However, for ≥128 sidecar processes, Reeber is equal to or faster than the 5 simulation time steps, such that the scalability of the entire code suite begins to decouple from that of Reeber alone. This behavior has different effects for the CS and CT in-transit configurations. For the CS configuration, the scalability of the code becomes asymptotically flat for ≥128 sidecar processes, because Reeber completes before the 5 simulation time steps are done. Any number of additional sidecar processes above ∼256 is wasted in this mode. For the CT configuration, the end-to-end wall clock time begins to *increase* for ≥128 sidecar processes, because even though Reeber is executing faster and faster, increasing number of sidecar processes decreases the number of simulation processes, which slows the entire code down.

In light of this behavior, it is critical to find the configuration which yields the best performance for in-transit analysis. Deviating from this configuration leads either to wasted compute resources, or to a degradation of overall code performance. We note that the optimal setup for Reeber in both the CS and CT configurations for this particular Nyx simulation - using 128 to 256 sidecar processes for Reeber and the remainder for simulation - is faster than running the entire code suite *in situ*. In particular, the CS mode with $2\text{,}048+256$ processes is faster than the *in situ* mode by ${\sim}30\%$ (${\sim}70~\mathrm{s}$). The CT mode is especially appealing, as it uses the same number of MPI resources as the *in situ* mode (2,048 processes total), and less than the corresponding CS configuration (2,176 to 2,304 processes).

Finally, we note that even the fastest post-processing configuration (256 sidecars in the ‘CS’ in-transit configuration) is still about ${\sim}35\%$ (${\sim}40~\mathrm{s}$) slower than running Nyx with no analysis at all. We identify at least two factors which contribute to this overhead cost. Firstly, the workload between the simulation MPI partition and the post-processing partition is not perfectly balanced; one is always waiting for the other. Secondly, when we run in-transit, we do not control the physical placement of MPI processes on the machine, whereas when running with no analysis, all processes do the same work and are more likely to be optimally placed together. Ideally, when post-processing, one would gather the compute partition as closely together as possible, and similarly for the post-processing partition. However, this is not guaranteed to occur, and indeed the data suggest that it indeed does not.

**Figure 9:**
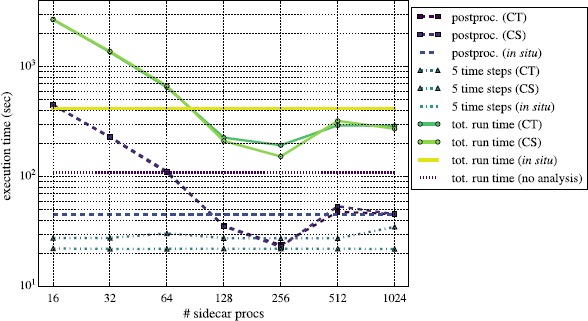
**Same as Figure** **8** **, except with Gimlet.** Time to post-processes for the CS and CT in-transit configurations are nearly identical.

Gimlet’s scaling in Figure 9, however, is more complex. This is because Gimlet performs a variety of tasks, calculating both PDFs, which strong scale very well, and power spectra, which do not. Specifically, while BoxLib can decompose the problem domain into an arbitrary block structure, the only domain decomposition strategy which FFTW3 supports is to stripe along the first dimension in row-major array indexing, or the last dimension in column-major indexing. (FAB data in BoxLib uses the latter.) Therefore, the Nyx problem domain must be divided into chunks which span the entire *x*-*y* plane. Furthermore, the maximum number of chunks we can create is the number of grid points of the domain along the *z*-axis (512 in this case). Since FFTW3 allows only one MPI process to work on each chunk, we are therefore limited to a total of 512 processes which can participate in the DFT calculation; the remaining processes (1,536 when running *in situ*) are idle. This load imbalance becomes worse as the problem size grows: if using ${\sim}{100\text{,}000}$ processes to simulate a $4\text{,}096^{3}$ grid, then ${\sim} {96\text{,}000}$ cores will be idle during the DFT calculation. This chunk decomposition problem has inspired the development of ‘pencil’-based decomposition strategies for 3-D DFTs which provide better scaling behavior (Habib et al. 2012; Habib et al. 2016). If we run Gimlet in-transit instead of *in situ*, however, we can address this problem by choosing a relatively small number of processes to participate in the DFT, leaving the rest to continue with the simulation. Similarly to the Reeber, case, when running with no analysis, Nyx is ${\sim}35\%$ faster than the best post-processing configuration (256 processes running in the ‘CS’ in-transit mode). We expect this gap to be the same because in the best CT configurations the code execution is limited by the simulation, and is independent of the post-processing algorithm.

The aggregate performance of Gimlet therefore represents a convolution of the scaling properties of both PDFs and power spectra. Although the power spectrum calculation scaling behavior quickly saturates as discussed above, we expect nearly ideal strong scaling behavior for the calculation of PDFs. Therefore, when we see in Figure 9 that the time for analysis *increases* between 256 and 512 analysis processes, this is due to the DFT calculations. In particular, when calculating the DFT of a 512^3^ grid on 512 MPI processes, each process has exactly one *x*-*y* plane of data. The ratio of work-to-communication may be low for such an extreme decomposition, leading to worse performance with 512 processes than with 256. Despite the jump in analysis time between 256 and 512 processes, however, the time decreases once again between 512 and 1,024 processes. When executing the DFT on 1,024 processes, we leave 512 idle, so the time for that component is likely exactly the same as it was with 512; in fact, the DFT calculation time will *never* decrease for >512 processes.^4^ The decrease in total analysis time is instead due to the continued strong scaling of the PDF calculations.

The relationship between the scalability of Gimlet and the entire code suite is different than for Reeber, chiefly because Gimlet execution takes significantly longer than does Reeber. For almost any number of sidecar processes in Figure 9, the time to execute Gimlet is longer than the corresponding 5 simulation time steps. (For 256 sidecar processes the two times are roughly equivalent.) As a result, Gimlet dominates the total code execution time, and the simulation+analysis strong scaling behavior follows that of Gimlet alone almost exactly.

As was the case with Reeber, we see in Figure 9 that some CS and CT configurations lead to faster end-to-end run times than *in situ*. Coincidentally, the threshold at which CT and CS runs become faster is between 64 and 128 sidecar processes, the same as Reeber. When using 256 sidecar processes, the CS configuration is ${\sim}65 \%$ faster than the *in situ* run, significantly larger than the 35% speedup seen with the CS mode when running Reeber. These values are functions both of the scalability of the analysis codes being used, as well as the total time they take to execute. The key point is that for both analysis codes, one can construct an in-transit MPI configuration which is significantly faster than running *in situ*.

**Figure 10:**
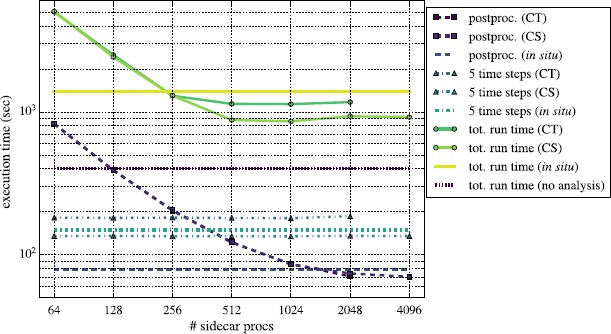
**Same as Figure** **8** **, except with Reeber running on the** $\pmb{1\text{,}024^{3}}$ **problem.** The short-dashed purple line indicates the Nyx run time with 4,096 MPI processes without performing any analysis.

**Figure 11:**
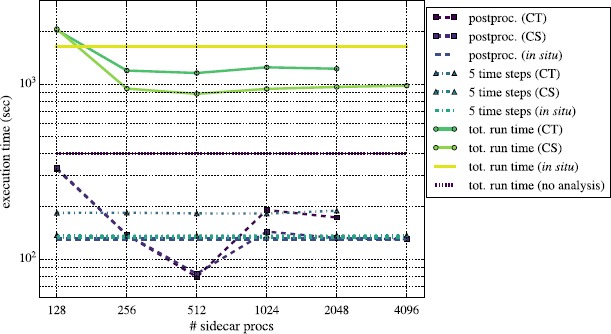
**Same as Figure** **9** **, except with Gimlet running on the** $\pmb{1\text{,}024^{3}}$ **problem.**

Figures 10 and 11 show results for the same analyses performed on a larger $1\text{,}024^{3}$ grid. When running with no analysis, we executed the code on 4,096 processes. In these tests we increased the number of MPI processes used in the *in situ* and CT in-transit configurations from 2,048 to 4,096, and the CS configuration used up to 8,192 total processes. The scaling behavior for Reeber running in all three modes is similar to that of the 512^3^ problem illustrated in Figure 8. When ≳512 processes are devoted to Reeber, it becomes faster than the 5 time steps of simulation, and the overall performance of the code plateaus, being bound by the simulation and not by post-processing. Gimlet running on the larger $1\text{,}024^{3}$ problem also scales similarly to that of the 512^3^ problem shown in Figure 9. The characteristic jump in post-processing wall clock time when going from 512 to 1,024 processes running Gimlet is due to the slowdown of FFTW when decreasing the number of *x*-*y* chunks from 2 per process to 1. (In Figure 9 the jump occurred when going from 256 to 512 processes.) Because the FFTW scales poorly, we also find, as in the smaller run, that most in-transit configurations are faster than running *in situ* since fewer processes are wasted computing the DFT. Reeber’s fastest in-transit configuration - CS with $2\text{,}048+1\text{,}024$ processes - is ${\sim}35\%$ faster than *in situ*. Gimlet’s fastest - CS with $2\text{,}048+512$ - is also ${\sim}35\%$ faster than the corresponding run *in situ*.

We note also that for these larger problems the gap between the fastest post-processing configurations and the case with no post-processing is wider than for the 512^3^ problem. For the 512^3^ problems it was about ${\sim}35\%$, but for these $1\text{,}024^{3}$ problems it is now about ${\sim}55\%$ (${\sim}500~\mathrm{s}$). This widening is likely due to exacerbation of the effects described above, particularly that the compute and post-processing MPI partitions are now spread even more widely across the machine, leading to larger MPI communication costs.

## Summary and prospects

*In situ* and in-transit data post-processing workflows represent a promising subset of capabilities which we believe will be required in order to be scientifically productive on current and future generations of computing platforms. They avoid the constraints of limited disk capacity and bandwidth by shifting the requisite data movement ‘upward’ in the architectural hierarchy, that is, from disk to compute node memory. One can imagine continuing this trend to even finer levels of granularity, shifting from data movement between compute nodes across an interconnect, to movement across NUMA domains within a compute node. This could be implemented in several different ways; one would be to use ‘thread teams’ introduced in OpenMP 4.0 (which is already widely implemented in modern compilers) to delegate simulation and analysis tasks within a compute node. A second approach would be to extend the MPI implementation we have introduced in this work to incorporate the shared memory ‘windows’ developed in MPI-3.

We have demonstrated the capability of these new *in situ* and in-transit capabilities in BoxLib by running two analysis codes in each of the two workflows. Although small in number, this sample of analyses - finding halos and calculation power spectra - is highly representative of post-processing tasks performed on cosmological simulation data. We caution, however, that the results presented in Section 5.4 are highly problem-dependent; although we found in-transit configurations which yield faster overall performance than *in situ* for both analysis codes, the situation may be different when running simulations on larger grids, using larger numbers of processes, running analysis algorithms with different scaling behavior, using a different frequency of analysis, etc. Furthermore, we highlight the caveat that, in some situations, on-the-fly data post-processing is not a useful tool, namely in exploratory calculations, which are and will continue to be critical components of numerical simulations. In these cases, other techniques will be more useful, including on-disk data compression. We anticipate, then, that a variety of tools and techniques will be required to solve these data-centric challenges in HPC.

Besides raw performance gains, *in situ* and in-transit workflows can improve the ‘time to science’ for numerical simulations in other ways as well. For example, by running both simulation and analysis at the same time, one eliminates an extra step in the post-processing pipeline. This reduces the chance for human error which can arise when one must compile and run two separate codes with two separate sets of inputs, parameters, etc.

The *in situ* and in-transit workflows we have discussed are not limited purely to floating-point applications, even though that has been our focus in this work. Another critical component of simulation is visualization, and recently both the ParaView and VisIt frameworks have implemented functionality for performing visualization on data which resides in memory (‘ParaView Catalyst’ (2016) and ‘libsim’ (2016)).

Our implementations of *in situ* and in-transit post-processing show that these types of workflows are efficient on current supercomputing systems: the expense of data movement via MPI in the latter workflow is, in the cases examined here, small compared to the total time spent performing simulation or analysis. Therefore, the penalty for trading disk space for CPU-hours (both of which are limited commodities) is not severe. While we have examined only two analysis codes in this work, in the future we will be able to evaluate the myriad other analysis workflows which are critical components of other BoxLib codes. Because we have built these capabilities into BoxLib itself, rather than into Nyx specifically, these workflows will support a wide variety of applications. The infrastructure described here will provide scientists working with BoxLib-based codes in astrophysics, subsurface flow, combustion, and porous media, an efficient way to manage and analyze the increasingly large datasets generated by their simulations.

## Footnotes

1 The Amr::CoarseTimeStep() function is the ‘outermost loop’ in the time-stepping algorithm which uses subcycling, i.e., the coarsest grids take the largest time steps (since they have the most lenient CFL condition), and the refined grids must take smaller time steps to satisfy their stricter CFL conditions. The procedure is hierarchical; each grid may take many more time steps than its coarser ‘neighbor,’ but ultimately all grids must synchronize at the end of the large time step taken by the coarsest level. If subcycling is disabled, then all grids, regardless of their level of refinement, take the same time step together. In the case of Lyman-*α* simulations, we do not use AMR, and so there is only one time step for all grids in each call to Amr::CoarseTimeStep(). The subcycling procedure in Nyx simulations which *do* use AMR is discussed in Almgren et al. (2013).

2 In the following text we refer to this simply as a ‘10-component grid.’

3 In production runs, one may wish to execute *both* analysis codes in a single simulation, but for the purposes of this performance study, we do not consider this case, as it obscures the performance behavior we seek to study.

4 For simulations in which the number of MPI processes calling FFTW3 is equal to or larger than the number of available chunks, we could select an arbitrarily smaller number of processes to perform the FFTW3 call, since it seems that using the maximum possible number of chunks and MPI processes does not lead to the fastest performance of the DFT. However, the optimal number of processes will likely be problem-dependent.

## References

[CR1] Agranovsky A (2014). Improved post hoc flow analysis via Lagrangian representations. 2014 IEEE 4th Symposium on Large Data Analysis and Visualization (LDAV).

[CR2] Almgren AS (2010). CASTRO: a new compressible astrophysical solver. I. Hydrodynamics and self-gravity. Astrophys. J..

[CR3] Almgren AS (2013). Nyx: a massively parallel AMR code for computational cosmology. Astrophys. J..

[CR4] Anderson L (2014). The clustering of galaxies in the SDSS-III baryon oscillation spectroscopic survey: baryon acoustic oscillations in the data releases 10 and 11 galaxy samples. Mon. Not. R. Astron. Soc..

[CR5] Bennett JC (2012). Combining in-situ and in-transit processing to enable extreme-scale scientific analysis. SC ’12 Proceedings of the International Conference on High Performance Computing, Networking, Storage and Analysis.

[CR6] Bleuler A (2015). Comput. Astrophys. Cosmol..

[CR7] BoxLib (2016). https://ccse.lbl.gov/BoxLib/index.html

[CR8] Bryan GL, Norman ML, Stone JM, Cen R, Ostriker JP (1995). A piecewise parabolic method for cosmological hydrodynamics. Comput. Phys. Commun..

[CR9] Colella P (1990). Multidimensional upwind methods for hyperbolic conservation laws. J. Comput. Phys..

[CR10] Colella P, Glaz HM (1985). Efficient solution algorithms for the Riemann problem for real gases. J. Comput. Phys..

[CR11] Davis M (1985). The evolution of large-scale structure in a universe dominated by cold dark matter. Astrophys. J..

[CR12] Frenk CS, White SDM, Bode P, Bond JR, Bryan GL, Cen R, Couchman HMP, Evrard AE, Gnedin N, Jenkins A, Khokhlov AM, Klypin A, Navarro JF, Norman ML, Ostriker JP, Owen JM, Pearce FR, Pen UL, Steinmetz M, Thomas PA, Villumsen JV, Wadsley JW, Warren MS, Xu G, Yepes G (1999). The Santa Barbara cluster comparison project: a comparison of cosmological hydrodynamics solutions. Astrophys. J..

[CR13] Frigo M, Johnson S (2005). The design and implementation of FFTW3. Proc. IEEE.

[CR14] Habib, S, et al.: The universe at extreme scale: multi-petaflop sky simulation on the BG/Q (2012). arXiv:1211.4864

[CR15] Habib S (2016). HACC: simulating sky surveys on state-of-the-art supercomputing architectures. New Astron..

[CR16] Haardt F, Madau P (2012). Radiative transfer in a clumpy universe. IV. New synthesis models of the cosmic UV/X-ray background. Astrophys. J..

[CR17] Heitmann K (2014). Large-scale simulations of sky surveys. Comput. Sci. Eng..

[CR18] Heitmann K (2015). The Q continuum simulation: harnessing the power of GPU accelerated supercomputers. Astrophys. J..

[CR19] Hockney RW, Eastwood JW (1988). Computer Simulation Using Particles.

[CR20] Hunter JD (2007). Matplotlib: a 2D graphics environment. Comput. Sci. Eng..

[CR21] Knebe A (2013). Structure finding in cosmological simulations: the state of affairs. Mon. Not. R. Astron. Soc..

[CR22] Lukić Z (2015). The Lyman *α* forest in optically thin hydrodynamical simulations. Mon. Not. R. Astron. Soc..

[CR23] Lukić Z, Reed D, Habib S, Heitmann K (2009). The structure of halos: implications for group and cluster cosmology. Astrophys. J..

[CR24] Mihalas D (1978). Stellar Atmospheres.

[CR25] Mo H, van den Bosch FC, White S (2010). Galaxy Formation and Evolution.

[CR26] Morozov, D, et al.: IsoFind: Halo finding using topological persistence (in preparation)

[CR27] Morozov D, Weber GH (2013). Distributed merge trees. PPoPP ’13: Proceedings of the 18th ACM SIGPLAN Symposium on Principles and Practice of Parallel Programming.

[CR28] Morozov D, Weber GH, Bremer PT, Hotz I, Pascucci V, Peikert R (2014). Distributed contour trees. Topological Methods in Data Analysis and Visualization III.

[CR29] Nouanesengsy B (2014). ADR visualization: a generalized framework for ranking large-scale scientific data using analysis-driven refinement. 2014 IEEE 4th Symposium on Large Data Analysis and Visualization (LDAV).

[CR30] Palanque-Delabrouille N (2013). The one-dimensional Ly*α* forest power spectrum from BOSS. Astron. Astrophys..

[CR31] ParaView catalyst for *in situ* analysis (2016). http://www.paraview.org/in-situ/

[CR32] Planck Collaboration (2014). Planck 2013 results. XVI. Cosmological parameters. Astron. Astrophys..

[CR33] Press WH, Schechter P (1974). Formation of galaxies and clusters of galaxies by self-similar gravitational condensation. Astrophys. J..

[CR34] Röpke FK (2006). Type Ia supernova diversity in three-dimensional models. Astron. Astrophys..

[CR35] Ross RB (2008). Visualization and parallel I/O at extreme scale. J. Phys. Conf. Ser..

[CR36] Sewell C (2015). Large-scale compute-intensive analysis via a combined in-situ and co-scheduling workflow approach. SC ’15 Proceedings of the International Conference for High Performance Computing, Networking, Storage and Analysis.

[CR37] Srisawat C (2013). Sussing merger trees: the merger trees comparison project. Mon. Not. R. Astron. Soc..

[CR38] Thielemann FK, Nomoto K, Yokoi K (1986). Explosive nucleosynthesis in carbon deflagration models of type I supernovae. Astron. Astrophys..

[CR39] Travaglio C (2004). Nucleosynthesis in multi-dimensional SN Ia explosions. Astron. Astrophys..

[CR40] Viel M (2013). Warm dark matter as a solution to the small scale crisis: new constraints from high redshift Lyman-*α* forest data. Phys. Rev. D.

[CR41] VisIt tutorial *in situ* (2016). http://www.visitusers.org/index.php?title=VisIt-tutorial-in-situ

[CR42] Williams S, Waterman A, Patterson D (2009). Roofline: an insightful visual performance model for multicore architectures. Commun. ACM.

